# Supplementary material for: Vaccination against the Protozoan Parasite Histomonas meleagridis Primes the Activation of Toll-like Receptors in Turkeys and Chickens Determined by a Set of Newly Developed Multiplex RT-qPCRs
Source: Vaccines (Basel). 2021 Aug 27;9(9):960. doi: 10.3390/vaccines9090960 (PMC8472887; doi:10.3390/vaccines9090960)
Supplement: Supplementary file 1 [file vaccines-09-00960-s001.zip › vaccines-1268763-supplementary.pdf]

Supplementary Table S1: Optimized set-up for multiplex RT-qPCR and concentrations of all candidate genes primers and probes are given with efficiency value for turkey and chicken species. For every candidate gene, 100nM of probe concentrations was used for all genes.

Set	gene symbol	primer concentrations (nM) for turkey	efficiency for turkey (%)	primer concentrations (nM) for chicken	efficiency for chicken (%)
1	*TLR 1A*	200	98.56	300	96.78
	TLR 2A	200		500	
	TLR 5	200		500	
2	*TLR 1B*	200	99.48	200	102.3
	TLR 2B	200		200	
	TLR 3	200		200	
	TLR 4	400		400	
3	*TLR 6*	600	96.75	N.A.	
	TLR 7	400			
	TLR 13	400			
4	*TLR 7*	N.A.		600	94.78
	TLR 21			400	
5	*IL1β*	400	97.73	400	97.34
	IL6	200		600	
6	*RPL 13*	300	98.72	500	99.12
	TFRC	400		400	

N.A.: Not applicable

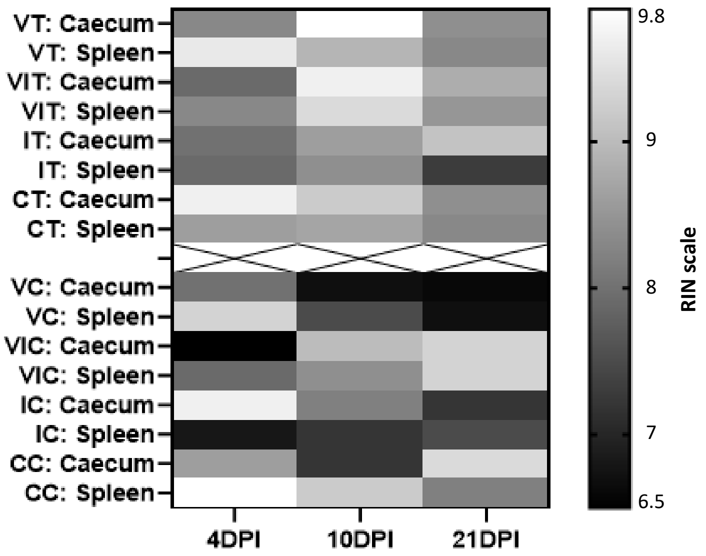

Supplementary Figure S1: Mean RNA integrity number (RIN) of each organ measured by Bioanalyzer 2100 (Agilent technologies) for all samples used in the present study.
